# Clinical decision support in the electronic health record: a primer for antimicrobial stewards and infection preventionists: work smarter so end users don’t work harder

**DOI:** 10.1017/ash.2024.448

**Published:** 2024-11-14

**Authors:** Mary Rochelle Smith, Julie J. Lee, Marisa Holubar, Jorge L. Salinas, Mindy M. Sampson, Richard J. Medford, Amy Chang

**Affiliations:** 1 Division of Infectious Diseases and Geographic Medicine, Stanford University School of Medicine, Palo Alto, CA, USA; 2 Division of Primary Care and Population Health, Stanford University School of Medicine, Palo Alto, CA, USA; 3 Division of Infectious Diseases & Geographic Medicine, Brody School of Medicine, East Carolina University, Greenville, NC, USA

## Abstract

**Objective:**

Computerized clinical decision support (CDS) assists healthcare professionals in making decisions to improve patient care. In the realms of antimicrobial stewardship (ASP) and infection prevention (IP) programs, CDS interventions can play a crucial role in optimizing antibiotic prescribing practices, reducing healthcare-associated infections, and promoting diagnostic stewardship when optimally designed. This primer article aims to provide ASP and IP professionals with a practical framework for the development, design, and evaluation of CDS interventions.

**Setting:**

Large academic medical center design: Established frameworks of CDS evaluation, “Five Rights” of CDS and the “Ten Commandments of Effective Clinical Decision Support”, were applied to two real-world examples of CDS tools, a Vancomycin Best Practice Advisory and a *Clostridioides Difficile* order panel, to demonstrate a structured approach to developing and enhancing the functionality of ASP/IP CDS interventions to promote efficacy and reduce unintended consequences of CDS.

**Conclusions:**

By outlining a structured approach for the development and evaluation of CDS interventions, with focus on end user engagement, efficiency and feasibility, ASP and IP professionals can leverage CDS to enhance IP/ASP quality improvement initiatives aimed to improve antibiotic utilization, diagnostic stewardship, and adherence to IP protocols.

## Clinical decision support

Computerized clinical decision support (CDS) aims to aid healthcare professionals in improving patient care. CDS interventions in antimicrobial stewardship (ASP) and infection prevention (IP) can streamline initiatives, improve antibiotic prescribing, and reduce healthcare-associated infections.^1,2^ However, thoughtful development of CDS interventions is essential for efficacy and minimizing unintended consequences.^1,3,4^ Utilizing established frameworks like the “Five Rights” of CDS and “Ten Commandments of Effective Clinical Decision Support” can maximize intervention utility.^5–7^ This article provides ASP and IP professionals examples for developing, designing, and assessing CDS interventions through real-world application involving a vancomycin Best Practice Advisory (BPA) and a *Clostridiodes difficile* (C diff) order panel.

## The CDS “five rights” in action

**Figure 1.**
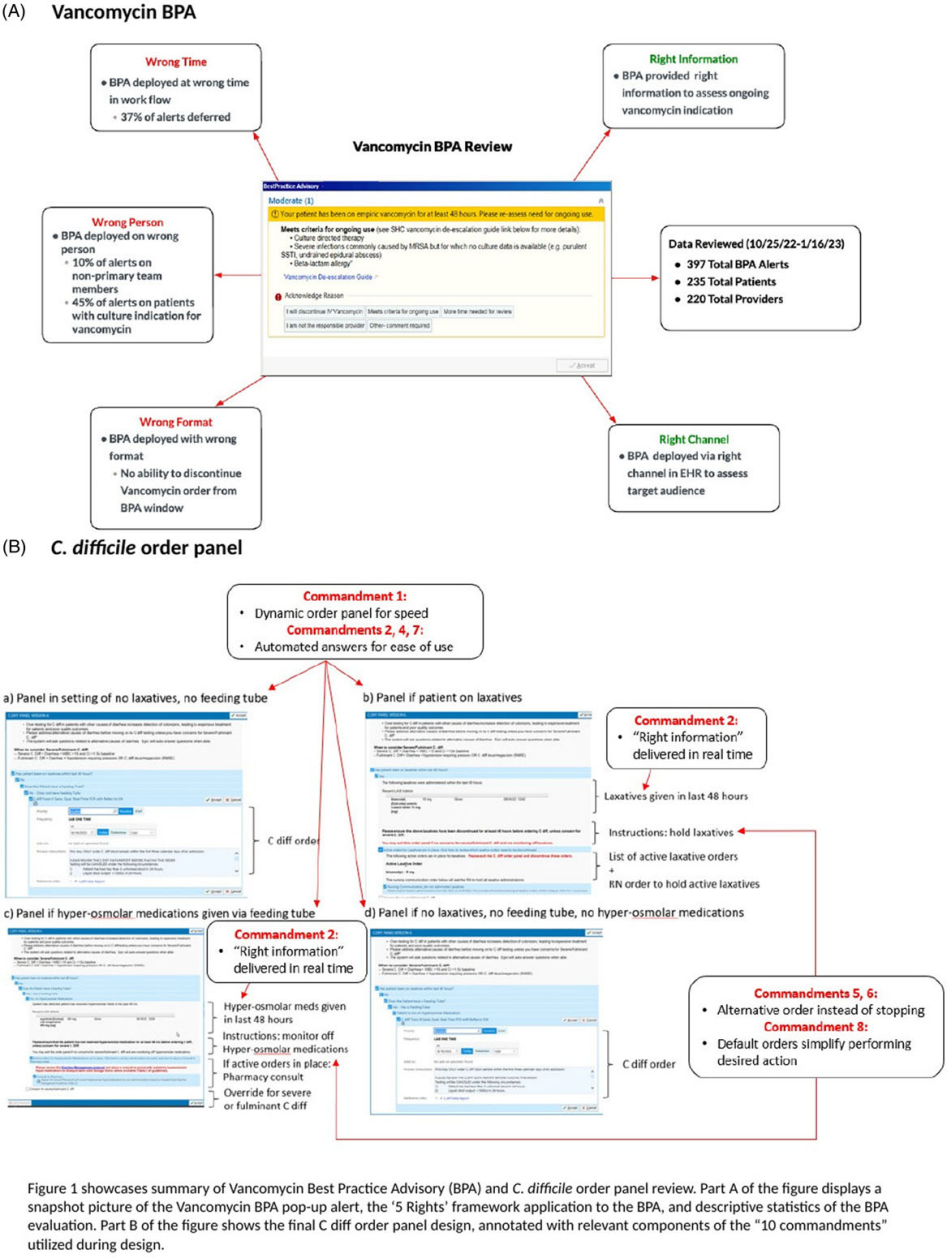
Summary of vancomycin BPA and *C. difficile* order panel.

The “Five rights” of CDS framework, based on the five rights of medication use, is a structured method for enhancing interventions to improve patient care outcomes^5,6^. Utilizing this framework, we assessed and enhanced our vancomycin BPA (Figure 1). This real-time interruptive pop-up alert acts as an “antibiotic timeout”, triggering primary clinicians to reassess empiric vancomycin orders 48–72 hours post-administration. This evaluation is outlined below:
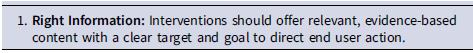



Our BPA design provided a clear reason for triggering, criteria for continued vancomycin use, and a link to institutional guidelines. No changes were implemented.
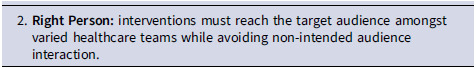



The patient target audience was those on vancomycin for ≥ 48 hours without a microbiologic indication, however 45% of BPA alerts fired for patients with microbiologic indication. Additionally, 10% of alerts fired for consultants/non-primary providers instead of primary clinicians.

We suppressed the BPA for patients with positive sterile site cultures for methicillin resistant *Staphylococcus aureus* within 72 hours. The BPA was also removed from ICU units where 50% of non-target prescriber interactions occurred.
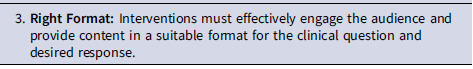



A BPA was the appropriate intervention format, as using interruptive alerts for this purpose has been supported by CDS literature.^8^ However, the original BPA design required providers to exit before they could discontinue the order. We added a ‘discontinue vancomycin’ button directly to the BPA to streamline the process.
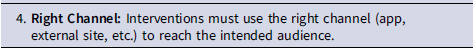



An alert within the electronic health record (EHR) was the appropriate channel, given this was the same application used to order vancomycin. No changes were made.
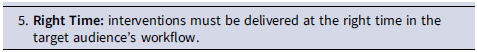



While the alert timing during vancomycin ordering seemed mostly appropriate, 37% of alerts were deferred, mainly in 6 ICU units where interruptions are common. Since alternative ASP interventions were in place, the BPA was removed from these units.

## “Ten commandments” in action

The “Ten Commandments” of effective CDS aim to reduce lag time and errors in evidence-based implementation^7^. We received a proposed design for an order panel, a collection of commonly grouped orders for user selection, with the goal to reduce inappropriate ordering of C diff by assessing alternative causes of diarrhea (Figure 1). We used the “Ten Commandments” to revise this design proposal, outlined below:
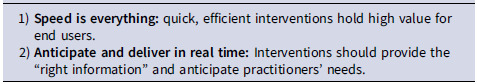



The initial proposed design requested the provider manually click through several questions/answers, reducing provider efficiency. To streamline, a dynamic component auto-answered questions using discrete EHR data.
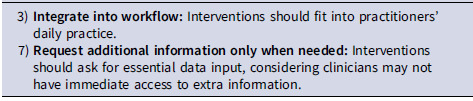



An order panel was deemed appropriate to provide CDS at time of ordering, but the original design had too many questions. Based on user feedback, the order panel was simplified to three questions where no extra information was required beyond automated components: (1) Is the patient on laxatives? (2) Does the patient have a feeding tube? (3) Is the patient on hyper-osmolar medications?
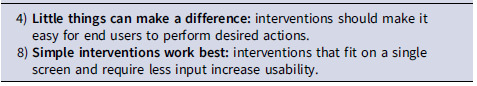



The order panel was designed to display active laxative/hyper-osmolar medications, providing users with the exact alternative cause of diarrhea. Careful design was used to reduce end user actions to one or two clicks only.
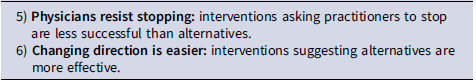



Rather than requesting the provider to stop ordering C diff, a nursing order was suggested to hold active laxatives, if present. For hyper-osmolar medications, the order panel suggested placing a pharmacy consult to use alternatives.
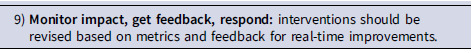



Requested metrics on order panel utilization revealed poor use due to a separate standalone historical *C. difficile* test order. This was eliminated to encourage order panel uptake.
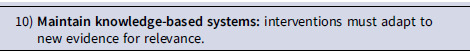



Ownership of the order panel was assigned to the requesting operational team to ensure timely updates with guideline changes.

## Conclusion

This report highlights the value of employing established frameworks to enhance the efficiency of CDS interventions for ASP/IP programs. ASP/IP best practices can sometimes create tension by supporting the development of barriers for providers to make it harder to do the wrong thing, but CDS literature suggests this may backfire.^7,9^ Our real-world examples illustrate that optimal design of CDS tools can both increase efficiency and align with best practices, making it easier for providers to do the right thing.
